# *cir-ITCH* Plays an Inhibitory Role in Colorectal Cancer by Regulating the Wnt/β-Catenin Pathway

**DOI:** 10.1371/journal.pone.0131225

**Published:** 2015-06-25

**Authors:** Guanli Huang, Hua Zhu, Yixiong Shi, Wenzhi Wu, Huajie Cai, Xiangjian Chen

**Affiliations:** 1 Department of Surgical Oncology, The First Affiliated Hospital of Wenzhou Medical University, Wenzhou, Zhejiang, P.R. China; 2 Departments of Obstetrics and Gynecology, The First Affiliated Hospital of Wenzhou Medical University, Wenzhou, Zhejiang, P.R. China; 3 Department of gastroenterology, The First Affiliated Hospital of Wenzhou Medical University, Wenzhou, Zhejiang, P.R. China; 4 Department of endoscopic surgery, The First Affiliated Hospital of Wenzhou Medical University, Wenzhou, Zhejiang, P.R. China; Medical College of Soochow University, CHINA

## Abstract

Noncoding RNAs (ncRNAs) are the dominant product of eukaryotic transcription. These products range from short microRNAs (miRNAs) to long intergenic noncoding RNAs (lincRNAs). Circular RNAs composed of exonic sequences represent an understudied form of ncRNA that was discovered more than 20 years ago. Using a TaqMan-based reverse transcriptase polymerase chain reaction assay, we analyzed the relationship between *cir-ITCH* expression and colorectal cancer (CRC) in a total of 45 CRCs and paired adjacent non-tumor tissue samples. We found that *cir-ITCH* expression was typically down-regulated in CRC compared to the peritumoral tissue. This result, as well as several follow-up experiments, showed that *cir-ITCH* could increase the level of *ITCH*, which is involved in the inhibition of the Wnt/β-catenin pathway. Therefore, our results showed that *cir-ITCH* plays a role in CRC by regulating the Wnt/β-catenin pathway.

## Introduction

Colorectal cancer (CRC) is a malignant neoplasm that is situated in the colon or rectum [1, 2]. CRC remains a major cause of cancer mortality in the developed world, largely due to its propensity to metastasize [3]. CRC poses a major public health problem, as it is the third most commonly diagnosed cancer in males and the second most common in females. There have been many comparative studies demonstrating epigenetic changes closely associated with the occurrence and development of CRC. Although the CRC research achievements are remarkable, the etiological factors and pathogenesis mechanisms underlying CRC development appear to be complex and heterogeneous.

Recently, circular RNA, a type of noncoding RNA that typically does not act by encoding a protein, was found to be closely related to tumor development [4]. Circular RNA is usually composed of more than one exon and is usually enriched with functional microRNA (miRNA) binding sites; for example, *CDR1* contains several conserved binding sites for miRNA-7 (miR-7). Recently, one study reported that *cir-ITCH* is a circular RNA that spans several exons of Ubiquitin (Ub) protein ligase (E3) (ITCH) [5–7]. The article indicated that *cir-ITCH* harbors many miRNA binding sites that can bind to the 3'-UTR of *ITCH*, including those for miR-7, miR-17, miR-214, miR-128 and miR-216b [5, 8]. The study of miRNAs is more mature than that of circular RNA; miRNAs are 21-nucleotide-long non-coding RNAs that have important roles in numerous biological processes in both plants and animals [9]. Mature miRNAs play important regulatory roles in cell growth, proliferation, differentiation, and cell death.

*cir-ITCH* plays an important role in the development and progression of ESCC [7]. *ITCH*’s targets, including p63, p73, and Notch1, are usually associated with tumor formation and chemosensitivity, demonstrating the connection of ITCH to cancer biology [10]. Previous research discussed circular RNA anti-cancer activities in malignant melanoma cell lines [11]. However, there are no reported studies on the functional roles of circular RNA in CRC.

In this study, we hypothesized that *cir-ITCH* plays a similar role in CRC. To test this hypothesis, we developed a method to delineate the transcriptional differences in *cir-ITCH* between CRC and paired adjacent non-neoplastic tissues.

## Materials and Methods

### Study subjects

All subjects in this study were homogenous Han Chinese. Forty-five CRC and corresponding paracancerous tissue samples were obtained from patients at The First Affiliated Hospital of Wenzhou Medical College (Wenzhou). There were no restrictions on the age, stage of CRC, sex or histology. At recruitment, each subject gave written informed consent by scheduling an interview at which they received a structured questionnaire. This study was approved by the institutional review board of Wenzhou Medical College. The clinical characteristics of all the patients are listed in Table 1.

**Table 1:** Baseline demographic and clinical characteristics of study populations.

Characteristics		population	
		N	(%)
**Age(years)**			
	≤40	4	(8.9)
	40–60	16	(35.5)
	≥60	25	(55.6)
**Sex**			
	Male	22	(48.9)
	Female	23	(51.1)
**Tumor invasion**			
	T1	5	(11.1)
	T2	9	(20)
	T3	31	(68.9)
**Family history**			
	Yes	6	(13.3)
	No	39	(86.7)
**Smoking**			
	Never	30	(66.7)
	Ever	15	(33.3)
**Drinking**			
	Never	21	(46.7)
	Ever	24	(53.3)
**Pathological type**			
	Highly	23	(51.1)
	Moderately	12	(26.7)
	Low	10	(22.2)
**Stage**			
	I	9	(20)
	II	16	(35.6)
	III	20	(44.4)

### Cell culture

The human CRC cell lines HCT116 and SW480 were purchased from the Cell Bank of Type Culture Collection of the Chinese Academy of Sciences, Shanghai Institute of Cell Biology and were passaged for less than 6 months.

### Construction of the circular RNA plasmid

We constructed the circular RNA plasmid used in this study. The construct method was previously published [8]. The resulting construct (*pcDNA3*.*1-cir-ITCH*) was verified by direct sequencing.

### RNA extraction and real-time quantitative polymerase chain reaction

Total RNA was isolated from cells and tissues using the TRIzol reagent according to the manufacturer’s instructions. The relative gene expression of *cir-ITCH* was determined using qPCR, which is based on the TaqMan method. *GAPDH* was used as an internal standard control, and all reactions were performed in triplicate [12, 13]. The primers used for qPCR amplification is the forward GCAGAGGCCAACACTGGAA, the reverse TCCTTGAAGCTGACTACGCTGAG, the probe CCGTCCGGAACTATGAACAACAATGGCA.

### RNase R digestion

The RNase R digestion reaction was performed following previously published procedures. The digestion and precipitation reactions were repeated twice with a ratio of 3 U of enzyme/mg of RNA [14].

### Transient transfections and luciferase assays

HCT116 and SW480 cells were transfected with the reporter plasmids described above using Lipofectamine 2000. Cells were co-transfected with the miRNAs according to the manufacturer’s instructions [15]. Each group included 6 replicates, and triplicate independent experiments were performed.

### Actinomycin D assay

HCT116 and SW480 cells were transiently transfected using Lipofectamine 2000 and were co-transfected with mRNAs as indicated for 24 h. The cells were then exposed to actinomycin D. The cells were harvested, and the stability of the *cir-ITCH* mRNA was analyzed using quantitative reverse transcription PCR (qRT-PCR). The assay was performed according to the previous article.

### Western blot

Western blot analysis to assess Wnt3a, β-catenin and β-action expression was carried out as described previously[16].

### Cell viability assay

The cell viability was measured using the Cell Counting Kit-8 (CCK-8) system according to the manufacturer’s instructions. There were 6 replicates for each group, and the experiments were repeated at least 3 times.

### Statistical analyses

The correlation between the expression of *cir-ITCH* and the *ITCH* gene in CRC tissues was determined using one-way analysis of variance and linear regression models. Differences between the groups were assessed by a paired, 2-tailed Student’s t-test. A *P*-value of <0.05 was considered statistically significant.

## Results

### Identification of the circular RNA

**Fig 1:**
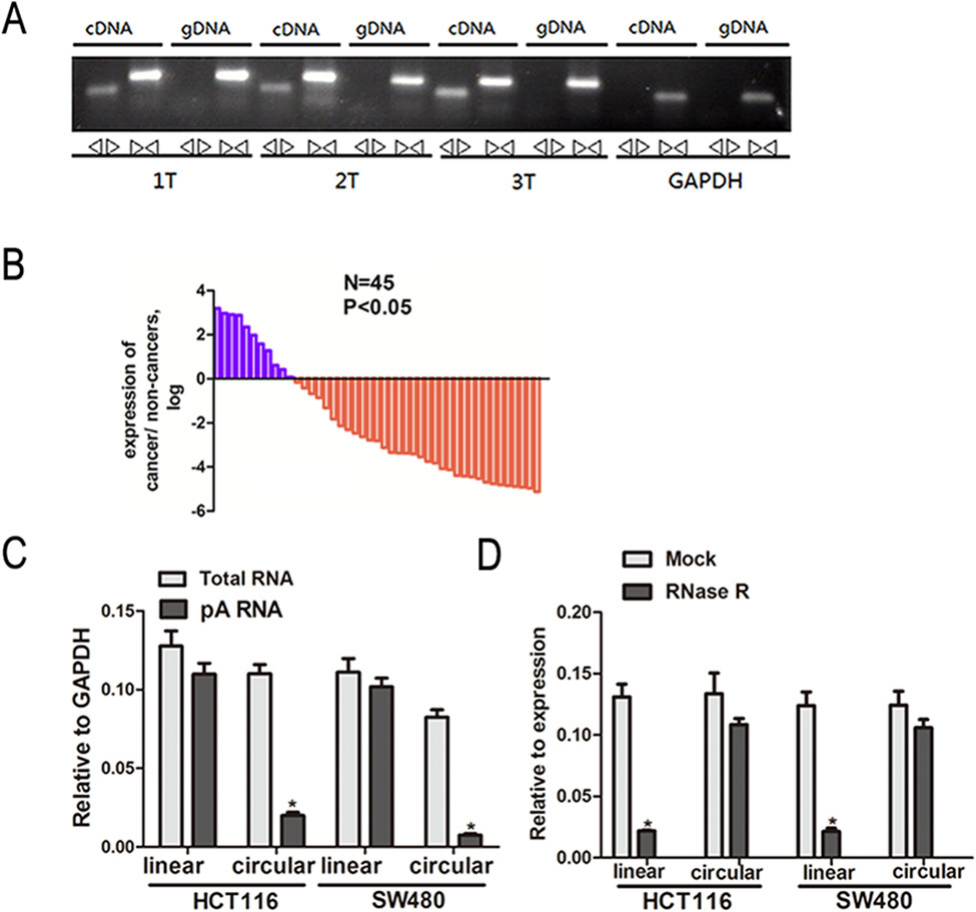
*cir-ITCH* is correlated with CRC. (A) Convergent primers can amplify circular RNAs and linear RNAs. Divergent primers amplify circular RNAs only in cDNA compared with genomic DNA (gDNA). GAPDH is linear control. (B) The *cir-ITCH* was expressed at a higher level in approximately 75.6% of the CRC adjacent tissues compared to match CRC tissues. The expression level of *cir-ITCH* was analyzed by qRT-PCR based on Taq-man and normalized to *GAPDH*. Data are represented as mean±SEM from three independent experiments. (C) Random primers and oligodT primers were used respectively in the reverse transcription experiments. The predicted *cir-ITCH* is absent in poly (A) enriched samples. (D) The predicted *cir-ITCH* is react against to RNase R treatment. 2-tailed student’s t-test were used in test the differences between groups *p < 0.05 compared to control.

Two sets of *ITCH* primers were designed for this study: a divergent set to amplify only the circular form and a convergent set to amplify the linear forms. We confirmed that the circular form was amplified using the divergent primers. cDNA and genomic DNA were used as the controls. As expected, no amplification was observed using the divergent primers on genomic DNA. GAPDH was used as the linear control (Fig 1A).

### Expression of *cir-ITCH* in CRC and the surrounding tissues

We used the divergent set of primers and utilized a TaqMan-based qRT-PCR assay to validate the presence of the circular RNA in 45 CRC tissues and paired non-cancerous tissues. *cir-ITCH* was expressed at a higher level in approximately 75.6% of the CRC tissues compared to that of the matched non-cancerous samples (Fig 1B).

### Characterization of *cir-ITCH* in CRC cells

We constructed a vector to express *cir-ITCH* in cells following the previous study and then performed experiments. The constructed plasmids were transiently transfected into HCT116 and SW480 cells.

In the reverse transcription experiments, we used random primers and oligo (dT) primers. The circular products were depleted in the polyA-enriched samples compared with the linear products. When using the oligo (dT) primers, the expression quantity (normalized to GAPDH) of linear *ITCH* was significantly higher than that of circular *ITCH* (Fig 1C) [11].

We used the enzyme RNase R, a highly processive 3'-5' exoribonuclease, to test our predictions of the circular RNA characteristics. This exonuclease degrades linear RNA molecules in a 3'-5' direction, but it does not work on circular RNAs [17, 18]. As expected, in contrast to the linear control RNAs, which were degraded, the predicted circular RNA was resistant to the RNase R treatment (Fig 1D).

### Interaction between *cir-ITCH* and miRNA

Recently, circular RNA has been identified as an abundant class of regulatory transcripts that function as miRNA sponges [5, 8]. The miRanda software was used to forecast the target miRNA. The sequence of the predicted microRNAs binding sites were presented in Table 2. We found that miR-7, miR-20a, and miR-214 can bind to the 3'-untranslated region (UTR) of *ITCH* and *cir-ITCH*. Therefore, we inserted the *ITCH* binding sequence into psiCHECK-2 and constructed luciferase reporters for these 3 miRNAs by transiently co-transfecting them into HCT116 cells. The construct had significantly reduced luciferase activity for miR-7, miR-20a, and miR-214 in a concentration-dependent manner in the control HCT116 cells (1 pmol miRNA-7: 1.02 ± 0.028 versus 1.236 ± 0.068, *P* = 0.05; 40 pmol miRNA-7: 0.808 ± 0.052 versus 1.236 ± 0.068, *P* = 0.02; 1 pmol miRNA-20a: 2.09 ± 0.123 versus 2.498 ± 0.068, *P* = 0.02; 40 pmol miRNA-20a: 1.887 ± 0.11versus 2.498 ± 0.068, *P* = 0.006; 1 pmol miRNA-214: 1.511 ± 0.059 versus 1.88 ± 0.043, *P* = 0.03; 40 pmol miRNA-214: 1.38 ± 0.058 versus 1.88 ± 0.043, *P* = 0.006). Similar results were found when we repeated the same experiments in the control SW480 cells.

**Table 2:** The sequence of the predicted miRNA binding sites on the the 3'-UTR region of *ITCH* and *cir-ITCH*.

microRNA	miRNA binding sites 3'-UTR	miRNA binding sites in *cir-ITCH*
miRNA-7	guggccacauguauaugucuuccc	ugagguaguagguuguauaguu
miRNA-214	uguauaugucuucccugcugu	acagcaggcacagacaggcagu
miRNA-20a	gauggacgugauauucgugaaau	uaaaggugcuuauagugcagguag

**Fig 2:**
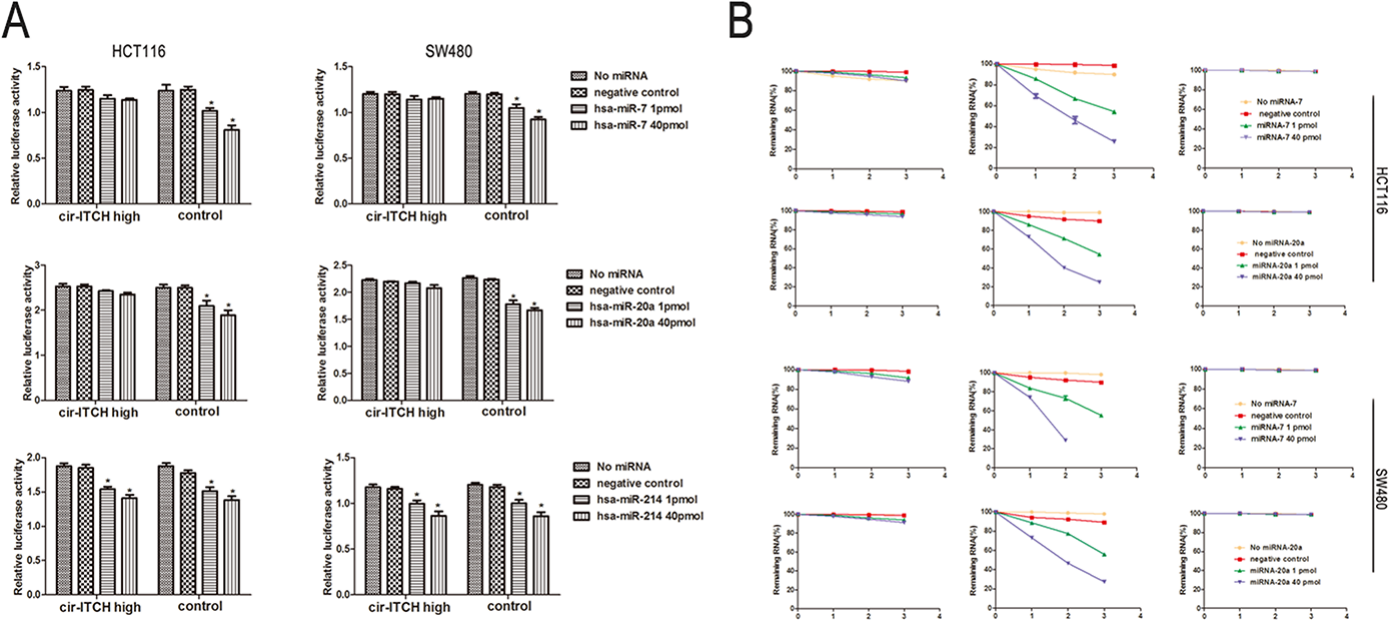
The sponges role of *cir-ITCH* in CRC cells. (A) Relative luciferase activity of the psiCHECK-2-ITCH constructs co-transfected with miR-20a, miR-7, miR-214 and inhibitor in HCT116 and SW480 cells. In *cir-ITCH* hyper-expression cells, there were no significant differences in luciferase activity when psiCHECK-2-*ITCH*-binding site with miRNAs were cotransfected into HCT116 and SW480 cells. Six replicates for each group and the experiment repeated at least three times. Data are mean±SEM. (B) HCT116 and SW480 cells after transfected with *cir-ITCH* and Control cells were respectively transfected with miR-20a, miR-7 and inhibitor for 24 h and were then further exposed to actinomycin D for 1, 2 and 3 h. Cells were harvested and the stability of *cir-ITCH* mRNA was analyzed by qRT-PCR relative to time 0 after blocking new RNA synthesis with actinomycin D; data are mean±SEM, normalized to *GAPDH*.

We performed the same experiment in *cir-ITCH* hyper-expression cells. The results showed that there were no significant differences in luciferase activity when the psiCHECK-2-*ITCH*-binding site with miR-7 and miR-20a was transfected into HCT116 and SW480 cells but that there was a significant difference for miR-214 (1 pmol miRNA-7: 1.147 ± 0.041 versus 1.236 ± 0.042, *P* = 0.069; 40 pmol miRNA-7: 1.138 ± 0.015 versus 1.236 ± 0.042, *P* = 0.12; 1 pmol miRNA-20a: 2.424 ± 0.017 versus 2.526 ± 0.058, *P* = 0.11; 40 pmol miRNA-20a: 2.345 ± 0.04 versus 2.526 ± 0.058, *P* = 0.069; 1 pmol miRNA-214: 1.539 ± 0.038 versus 1.877 ± 0.039, *P* = 0.001; 40 pmol miRNA-214: 1.407 ± 0.051 versus 1.877 ± 0.039, *P* = 0.004) (Fig 2A).

HCT116 and SW480 cells transfected with constructed plasmid were treated with actinomycin D in the presence of 1 or 40 pmol of miR-7 and miR-20a, and the total RNA was harvested at the indicated time points. The results show that in the control cells, the *cir-ITCH* levels remained at 20–30%; however, there was little change in the *cir-ITCH* levels in the HCT116 cells following incubation with actinomycin D. We repeated these experiments in SW480 cells with the same results (Fig 2B).

### Association of *cir-ITCH* and *ITCH* in CRC

**Fig 3:**
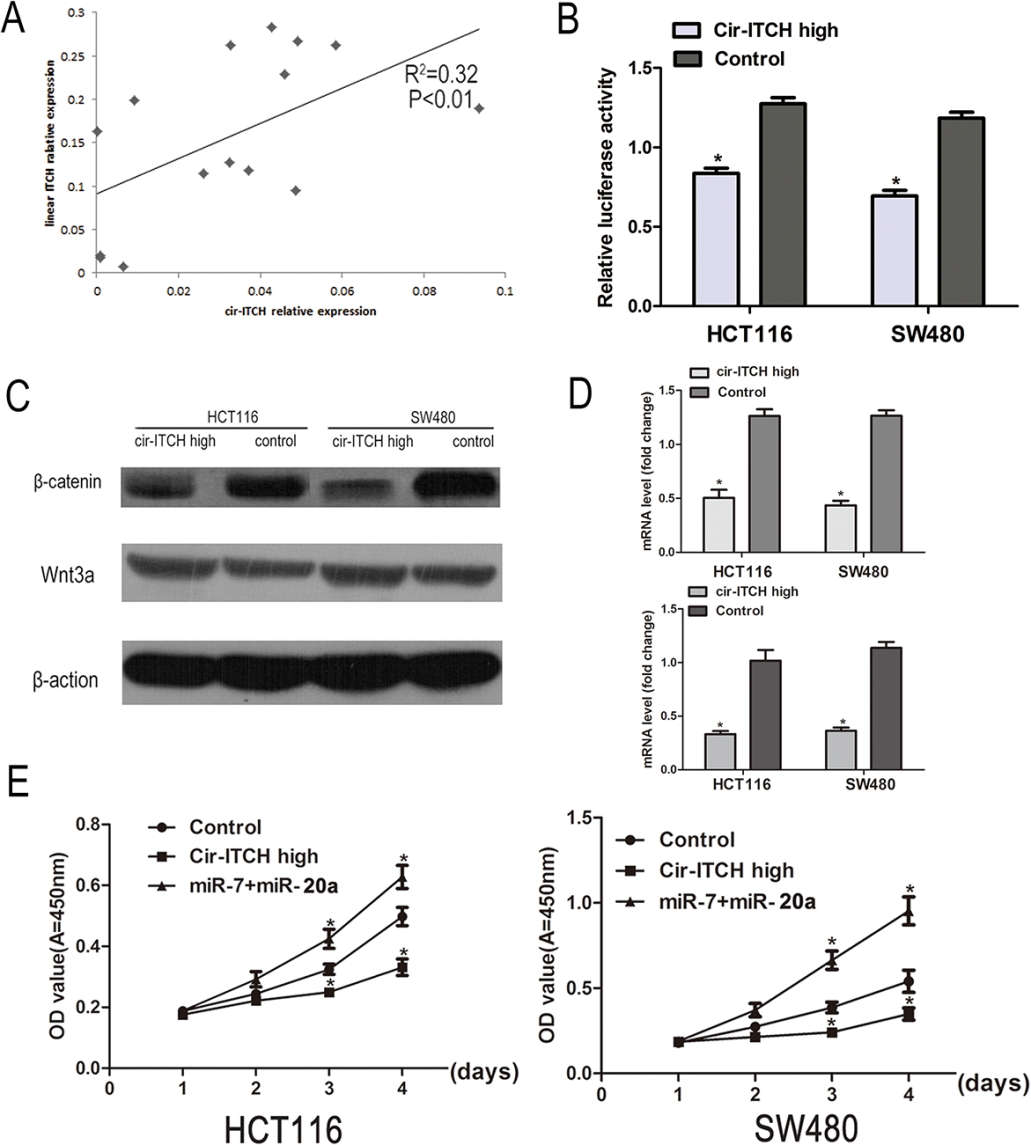
*cir-ITCH* involves in the regulation Wnt/b-catenin signaling pathway in vivo. (A) The linear correlations between the *cir-ITCH* expression levels and linear *ITCH* were tested. The relative expression value was normalized by *GAPDH* expression level. (B) A TCF luciferase reporter assay was performed. The luciferase activity was normalized to the Renilla luciferase activity. (C) The protein levels of Wnt3a and β-catenin was assessed in CRC cells (HCT116 cells and SW480 cells) by Western blot. (D) The mRNA level of *c-myc* and *cyclinD1*was detected by quantitative RT-PCR after transfected with *cir-ITCH* or Control cells in CRC cells. (the upper is *c-myc* and the lower is *cyclinD1*) Data are mean±SEM and representative of three independent experiments. (E) HCT116 and SW480 cells were seeded in 96-well plates after been transfected, and cell proliferation was performed daily for 3 days using the CCK-8 assay. Six replicates for each group and the experiment repeated three times. Data are mean±SEM. *P<0.05 compared with controls.

Recently, *cir-ITCH* was reported to regulate gene expression through its role as an miRNA sponge, thus protecting the miRNAs’ target genes. Therefore, we tested the correlation between *cir-ITCH* and *ITCH* in an additional 15 pairs of CRC adjuvant non-cancerous tissues. The results showed that patients with higher *cir-ITCH* expression levels in the CRC tissues had a substantial up-regulation of linear *ITCH* (R^2^ = 0.32, *P* < 0.01; Fig 3A).

### *cir-ITCH* is involved in the regulation of the Wnt/β-catenin signaling pathway in vivo

ITCH protein ubiquitinates the phosphorylated form of Dvl2 and promotes its degradation, thereby inhibiting canonical Wnt signaling [19]. To further verify whether *cir-ITCH* regulates the Wnt/β-catenin signaling pathway in CRC cells, we used a β-catenin/TCF-responsive luciferase reporter assay. The results showed that overexpression of ITCH inhibited the TOP flash activity in a dose-dependent manner (Fig 3B). The β-catenin and Wnt3a levels were analysised using western blot in cells with *ITCH* hyperexpression, and as shown in Fig 3C, there was an obvious decrease in β-catenin levels while there were no change in Wnt3a expression. We then tested the expression of *c-myc* and *cyclinD1*, two target genes of the Wnt/β-catenin signaling pathway, in cells transfected with *cir-ITCH*. Their expression was also suppressed (Fig 3D).

### *cir-ITCH* modulates cell growth

We performed CCK-8 assays to test the effects of *cir-ITCH* on cell proliferation in CRC cells. We observed a consistent decrease in the cell proliferation of HCT116 (28%-39% decrease) and SW480 cells (15%-33% decrease) when *cir-ITCH* was overexpressed at physiological levels through the CCK-8 assays compared to that of the control (Fig 3E).

## Discussion

In this study, we identified *cir-ITCH* in CRC and found that it was dramatically down-regulated in CRC tissues using a TaqMan-based reverse transcriptase polymerase chain reaction assay, indicating the potential function of *cir-ITCH* in CRC. A series of experiments illustrated the correlation between *cir-ITCH* and miRNAs, demonstrating that *cir-ITCH* plays an important role as an miRNA sponge in the process of CRC development.

Thousands of lincRNAs have been identified in humans and mice, and many are closely related to the development of various types of cancer, such as esophageal squamous cell carcinoma (ESCC) and endometrial carcinoma [20, 21]. Circular RNA is one type of lincRNA that plays a role in tumor development. To our knowledge, *ITCH* is one of the E3 ubiquitin protein ligases that specifically targets p73 [22], Dvl2 [19], and Notch1 [23], all of which are usually associated with tumor formation and chemosensitivity.

Although the functions of miRNAs are far from being fully understood, it is predicted that approximately 30% of protein-encoding genes are controlled by miRNAs[24], and some studies have suggested that miRNA can bind to the 3'-UTR of *ITCH* to decrease its expression [25]. Memczak et al. (2013) and Hansen et al. (2011) proposed that the cerebellar degeneration-related protein 1 (CDR1) locus harbors 70 conserved matches to the miR-7 seed. This striking feature suggested that circular RNA has a possible function as an miRNA sponge [5, 8, 26]. Previous research has shown that *cir-ITCH* acts as a sponge for miR-7, miR-17, and miR-214, whereas in the present study, we found that in the CRC cell lines, miR-7 and miR-20a down-regulate *ITCH* expression by binding to its 3'-UTR. Further, these miRNAs are always harbored by *cir-ITCH*. In our study, *cir-ITCH* did not act as a sponge for miR-214. To our knowledge, ectopic expression of miR-214 suppresses proliferation, migration, and invasion in vitro, whereas miR-214 knockdown promotes proliferation, migration, and invasion in CRC cell lines [27].

miRNAs have long been associated with cancer. Elevated miR-7 expression has been described in a variety of tumor types, including CRC, and has been implicated in oncogenesis, classification, and cancer progression [28]. miR-20a contributes to the increased proliferation and invasiveness of CRC cells [29]. Based on this information, our data verify our predecessors' work. From the above data, it was shown that *cir-ITCH* participates in the development of CRC through regulation of miRNA activity.

Aberrant regulation of the Wnt signaling pathway has emerged as a prevalent theme in cancer biology, and it is crucial for the onset and progression of human CRC. Approximately 90% of sporadic colon cancers show aberrant Wnt signaling [30]. *cir-ITCH* acts as an miRNA sponge and increases the level of *ITCH*, which is involved in the Wnt/β-catenin pathway. According to previous research, ITCH can promote the ubiquitination and degradation of phosphorylated Dvl2 and, therefore, inhibit canonical Wnt signaling [19]. A β-catenin/TCF-responsive luciferase reporter assay was used to examine whether a single gene regulates the Wnt/β-catenin signaling pathway. Our study and other previous results reported that overexpression of *cir-ITCH* significantly suppressed the relative TCF transcriptional activity.

The oncogenes *c-myc* and *cyclinD1* are effector proteins of the karyomitosis signal, which can trigger and regulate the transcription of genes related to proliferation. They are frequently overexpressed in several human tumors, including CRC [31]. Our results showed that in cells transfected with *cir-ITCH*, the expression of *c-myc* and *cyclinD1* was markedly decreased. Therefore, we suggest that *cir-ITCH* has an inhibitory effect on the canonical Wnt pathway. *cir-ITCH* plays an anti-tumor role by controlling miRNA activity, and it has an inhibitory role in the canonical Wnt pathway, inhibiting *c-myc* and *cyclinD1* expression.

In summary, our present study indicates that the miRNA sponge *cir-ITCH* represents a new generation of technology for miRNA inhibition. *cir-ITCH* is involved in the Wnt/β-catenin pathway, and by inhibiting this pathway, it plays a role in CRC.
